# Conversion of Whey Protein Aerogel Particles into Oleogels: Effect of Oil Type on Structural Features

**DOI:** 10.3390/polym13234063

**Published:** 2021-11-23

**Authors:** Stella Plazzotta, Isabella Jung, Baldur Schroeter, Raman P. Subrahmanyam, Irina Smirnova, Sonia Calligaris, Pavel Gurikov, Lara Manzocco

**Affiliations:** 1 Department of Agricultural, Food, Environmental and Animal Sciences, University of Udine, Via Sondrio 2/A, 33100 Udine, Italy; stella.plazzotta@uniud.it (S.P.); lara.manzocco@uniud.it (L.M.); 2 Institute of Thermal Separation Processes, Hamburg University of Technology, Eißendorfer Straße 38, 21073 Hamburg, Germany; isabella.jung@tuhh.de (I.J.); baldur.schroeter@tuhh.de (B.S.); raman.subrahmanyam@tuhh.de (R.P.S.); irina.smirnova@tuhh.de (I.S.); 3 Laboratory for Development and Modelling of Novel Nanoporous Materials, Hamburg University of Technology, Eißendorfer Straße 38, 21073 Hamburg, Germany; pavel.gurikov@tuhh.de

**Keywords:** aerogel, oleogel, rheological properties, oil type, absorption

## Abstract

Protein aerogel particles prepared by supercritical-CO_2_-drying (SCD) of ground whey protein (WP) hydrogels (20% *w*/*w*, pH 5.7) were converted into oleogels by dispersion in selected edible oils (castor, cod liver, corn, flaxseed, MCT, peanut and sunflower oil). The obtained oleogels were analysed for oil content, microstructure, rheological properties, and ATR-FTIR spectra. Except for castor oil, solid-like, plastic materials with comparable composition (80% oil, 20% WP) and rheological properties (G′~3.5 × 10^5^ Pa, G″~0.20 × 10^5^ Pa, critical stress~800 Pa, tanδ~0.060) were obtained. Optical and confocal microscopy showed that the generated structure was associated with the capillary-driven absorption of oil into the porous aerogel particles interconnected via particle-particle interactions. In this structure, the oil was stably entrapped. Results evidenced the reduced role of edible oil characteristics with the exception of castor oil, whose high polarity probably favoured particle–oil interactions hindering particle networking. This work demonstrates that WP aerogels could be regarded as versatile oleogel templates allowing the structuring of many edible oils into solid-like materials.

## 1. Introduction

The development of oleogels, i.e., gels in which a continuous liquid oil phase is immobilised into a supramolecular network of self-assembled molecules [1], has attracted much research interest in the last decade as a feasible strategy to reduce saturated and trans fats content [2,3,4]. Recently, these systems have also been proposed as functional components able to modulate lipolysis as well as the delivery of bioactive lipophilic molecules [5,6,7].

Up to now, numerous approaches to oil structuring have been proposed. The most widely studied method relies on the direct dispersion of one or more lipophilic gelators (e.g., saturated monoglycerides, waxes, ethylcellulose, mixtures of phytosterols-sterol esters) into liquid oil, followed by heating above the gelator melting temperature and subsequent controlled cooling which allows the oleogelator to self-assemble into oil [8]. Although this operational simplicity, the use of liposoluble oleogelator candidates presents some issues. Most of them have to be listed as food additives on food labels. Thus, from one side, they are subjected to strict food regulations, and from the other side, they are increasingly avoided by food industries with a view of offering consumers clean label products [9]. Moreover, the heating phase required for oil structuring can trigger oil oxidation with a consequent quality and nutritional depletion [10].

Indirect oleogelation is under study as an alternative “cold” solution to entrap oil into a polymeric network made of hydrocolloids, such as carbohydrates and proteins [9,11]. Due to their hydrophilic nature, the entrapment of oil is challenging, but highly required by food industries offering foods with improved health functionalities and clean labels. Among indirect oleogelation strategies, the dried-template approach implies the initial gelation of the selected hydrocolloid with the formation of a hydrogel and the subsequent controlled removal of water. Finally, the dried scaffold is impregnated with oil leading to the formation of the oleogel [12,13]. Both polysaccharide (e.g., starch, κ-carrageenan) and protein (e.g., egg, whey proteins) dried templates are good candidates for the production of oleogels via the dried-template approach [13,14,15,16,17,18,19]. Based on the literature available, the structure of the scaffold is the pivotal factor steering the oleogel characteristics. Depending on the drying technique applied (e.g., air-drying, freeze-drying, supercritical-CO_2_-drying) the polymeric network structure could deeply change, leading to different oil absorption capacities [19]. While air-drying and freeze-drying commonly lead to the loss of the original gel structure, supercritical-CO_2_-drying allows for the preservation of polymeric network, by avoiding the formation of liquid–vapour interfaces and capillary tensions [20,21,22,23].

In this context, aerogels prepared via supercritical-CO_2_-drying have been indicated as particularly challenging dried materials able to generate oleogels [13,14,24]. Aerogels are defined as a special type of nanostructured materials formed by bonded particles or nanometric fibres obtained from a gel by removing the pore fluid and endowed with special physical features [19,25]. Solid nature, low bulk density, open porosity (usually in the 95–99.99% range) and high specific surface areas (up to 1200 m^2^/g) stand out as specific physical properties that the material should fulfil to fit in the consensual definition of aerogel.

In our previous research, we have demonstrated that supercritical-CO_2_-dried whey protein (WP) aerogel particles are particularly interesting as templates for sunflower oil structuring [26]. The aerogel particles were obtained by supercritical-CO_2_-drying of ground WP monolithic hydrogels, resulting in a white powder with low density (0.21 g/cm^3^) and a microscopic structure characterised by a fine porous network (pore size < 1 µm). Upon dispersion in sunflower oil, a plastic and deformable oleogel was obtained, whose peculiar rheological properties are due to the ability of supercritical-dried particles to absorb oil in their porous structure and establish inter-particle hydrophilic interactions, forming a network.

The proposed approach is promising but, at the moment, tested only with sunflower oil. It can be inferred that the networking capability of WP aerogels in the presence of oil could be highly affected by oil characteristics. The present study aimed to explore the possibility to prepare oleogels from WP aerogels by using different oils and to evaluate the structural properties of the obtained systems. To this aim, WP protein aerogel powder was prepared by supercritical-CO_2_-drying (SCD), as described in our previous work [26] and used to prepare oleogels by absorption of edible oils (castor, cod liver, corn, flaxseed, MCT, peanut and sunflower oil), with different fatty acid composition, viscosity, and polarity. The obtained oleogels were analysed for oil content, microstructure, rheological properties, and ATR-FTIR spectra. This study demonstrates that WP aerogels could be regarded as versatile oleogel templates allowing the structuring of many edible oils into solid-like materials.

## 2. Materials and Methods

### 2.1. Materials

Whey protein isolate (94.7% protein content; 74.6% β–lactoglobulin, 23.8% α–lactabumin, 1.6% bovine serum albumin) was purchased from Davisco Food International Inc. (Le Sueur, MN, USA). Castor, cod liver, corn, flaxseed, peanut, and sunflower oil were purchased in a local market. MCT 60-40 (MCT with fatty acid composition: C6:0 ≤ 0.5%, C8:0 55–66% and C10:0 35–45%) was purchased from Cremer Oleo Division (Hamburg, Germany). All solvents were purchased from Sigma-Aldrich (Milan, Italy). Deionized water (System advantage A10^®^, Millipore S.A.S, Molsheim, France) was used for all the analyses.

### 2.2. Preparation of Oleogels

WP aerogel particles were prepared as described by Plazzotta et al. [26]. Briefly, WP isolate aqueous solutions (20% *w*/*w*) were adjusted at pH 5.7 and gelled at 85 °C for 15 min. The obtained hydrogel was cooled and homogenized by using a high-speed mixer at 13,000 rpm for 3 min (Polytron PT-MR3000, Kinematica AG, Littau, Switzerland). The hydrogel was then dispersed in ethanol (0.1 g/mL), homogenized and collected by centrifugation at 13,000 *g* for 10 min at 4 °C (Avanti J-25, Beckman, Palo Alto, CA, USA). This procedure was repeated twice to completely remove water. The alcolgel particles were dried at a temperature of 60 °C, pressure of 120 bar, under a continuous flow of CO_2_ (flow rate = 120–160 g/min) through the autoclave until complete extraction of EtOH was achieved after 6 h. The dried particles were ground 1 min using a domestic grinder (MC3001, Moulinex, Milan, Italy) and dispersed into oil (0.1 g/mL), homogenized and collected by centrifugation, as previously described. This procedure was repeated twice, obtaining the oleogels.

### 2.3. Oil Viscosity

Oil viscosities were measured using a RS6000 Rheometer (Thermo Scientific RheoStress, Haake, Germany), equipped with a Peltier system for temperature control. The experiments were performed using concentric cylinder geometry, and measurements were carried out at 20 °C. The shear rate was increased step-wise from 0.3 to 120 s^−1^. All the oils considered showed a Newtonian behaviour.

### 2.4. Image Acquisition

Sample images were acquired using an image acquisition cabinet (Immagini & Computer, Bareggio, Italy) equipped with a digital camera (EOS 550D, Canon, Milano, Italy). The light was provided by four 23 W frosted photographic floodlights, in a position allowing minimum shadow and glare.

### 2.5. Optical Microscopy

Samples were gently placed on a glass slide, covered with a cover slide and observed using a Leica DM 2000 optical microscope (Leica Microsystems, Heerbrugg, Switzerland). The images were taken at 200× magnification using a Leica EC3 digital camera (Leica Microsystems, Heerbrugg, Switzerland) and imported in jpeg format with the Leica Suite Las EZ software (Leica Microsystems, Heerbrugg, Switzerland).

### 2.6. Scanning Electron Microscopy

The pore structure of the aerogels was characterized via scanning electron microscopy (SEM) (SE2-detector, Zeiss Supra VP55, Jena, Germany). Samples were sputtered with a thin layer of gold (ca. 7 nm, Sputter Coater SCD 050, BAL-TEC) before analysis was started. The measurements were carried out under high vacuum at an accelerating voltage of 4 kV and a working distance of 3.3 mm.

### 2.7. Confocal Microscopy

A 0.2% aqueous solution of Fast Green and Nile Red (Sigma Aldrich, Milan, Italy) was used to stain, respectively, the proteins and the oil of the oleogel samples. After staining, the samples were gently mixed by hand, placed on the microscope slide, covered with a cover slide and observed using a confocal laser scanning microscope at 100× magnification (Leica TCS SP8 X confocal system, Leica Microsystems, Wetzlar, Germany). Images were imported in jpeg format using the software LasX 3.5.5 (Leica Microsystems, Wetzlar, Germany).

### 2.8. Specific Surface Area and Pore Size

Characterization of aerogels microstructural properties was carried out by low-temperature N_2_ adsorption-desorption analysis (Nova 4000e Surface Area Analyzer, Quantachrome Instruments, Boynton Beach, FL, USA). An overall sample mass of approx. 20 mg was used, and all samples were degassed under vacuum at 60 °C for at least 4 h before each analysis. The Brunauer–Emmett–Teller (BET) method was used to determine the specific surface area. The pore volume of the mesopores and the mean pore diameter of mesopores were determined by the Barrett–Joyner–Halendia (BJH) method.

### 2.9. Specific Density

The specific density (g/cm^3^) of the aerogel particles was estimated by weighing 1 mL of dried material in a graded cylinder.

### 2.10. Oil Content

The oil content was determined according to the AOAC Official Method 2003.05, i.e., the Soxtec modification of the Soxhlet solvent extraction procedure [27].

### 2.11. Rheological Analysis

The viscoelastic properties (moduli G′, G″ and tan δ) were tested using a RS6000 Rheometer (Thermo Scientific RheoStress, Haake, Germany), equipped with a Peltier system for temperature control. Measures were performed using a parallel plate geometry at 20 °C with a gap of 2.0 mm. Oscillatory sweep tests to identify the linear viscoelastic region (LVR) were performed increasing stress from 1.0 to 1.0 × 10^4^ Pa at 1 Hz frequency. Critical stress (Pa) was identified as the stress value corresponding to a 10% drop in G′ value. Frequency sweep tests were then performed increasing frequency from 0.1 to 20 Hz at stress values selected in the LVR.

### 2.12. Data Analysis

Determinations were expressed as the mean ± standard error of at least three repeated measurements from two experiment replicates. Statistical analysis was performed by using R ver. 3.0.2 (The R Foundation for Statistical Computing). A one-way analysis of variance (ANOVA) was carried out. Significantly different samples were determined using the Tukey test (*p* < 0.05).

## 3. Results

### 3.1. Aerogel Particle Properties

**Figure 1:**
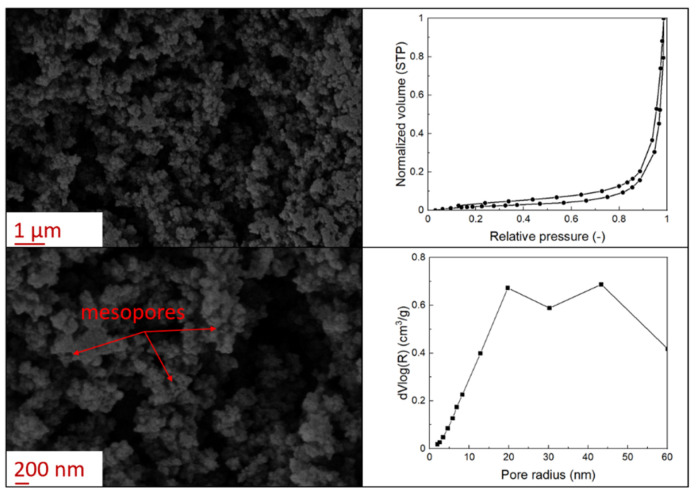
SEM pictures of aerogel microstructure (**left**), nitrogen adsorption–desorption isotherm (**top right**), pore size distribution calculated by BJH method (**bottom right**).

The supercritical drying of ground whey protein alcogels resulted in the formation of an aerated white powder, showing a low density (0.2 g/cm^3^). The N_⁠2_ adsorption-desorption isotherm (Figure 1, top right) of the WP aerogels shows a type IV hysteresis, which is characteristic for mesoporous materials with macroporous content. The pore size distribution is very broad (Figure 1, bottom right), with maxima in the range of larger mesopores and pore diameters which are reaching the macroporous range. It is evident from the SEM pictures (unprocessed pictures are reported in the Supplementary Figure S1), that a mainly macroporous, agglomerated structure was obtained, whereas primary aggregates contain the mesopores. The specific surface area and the mesoporous volume (V_meso_) are therefore comparatively low (65 m^2^/g; 0.54 cm^3^/g). An estimation of the overall macroporous volume via V_macro_ = 1/0.2 g/cm^3^-V_meso_ yields V_macro_ = 4.5 cm^3^/g and a ratio of V_macro_/V_meso_ of approx. 9:1. While the high macroporous content provides an overall low density and high porosity of the material, an increase of the mesoporous fraction would be necessary in order to obtain higher specific surface areas.

Mechanistically, the obtained microstructure is related to the unfolding behaviour of the proteins globules during the gelation step. Since the protein structure is well stabilized at conditions close to the isoelectric point, unfolding is limited and non-covalent interactions like electrostatic, hydrogen and van der Waals interactions play a more pronounced role, resulting in less fine stranded, agglomerated and denser microstructures [28]. This is generally reflected by the BET-surface areas of protein aerogels (including WP), which are strongly related to the pH-value during the gelation step and have been documented to cover a wide range (approx. 10–400 m^2^/g) [20,28,29]. Results in this work are therefore in the expected range for gels produced at pH = 5.7.

### 3.2. Oil Properties

Oils were selected as different in fatty acid composition, viscosity and dielectric properties. These characteristics have been demonstrated to significantly impact the structure of oleogels prepared with other gelators [30,31,32,33,34]. MCT was considered since composed by short-chain triacylglycerols and thus characterised by a low viscosity (Table 1). The other considered oils were mainly composed by long-chain fatty acids with different unsaturation degree, accounting for comparable viscosity (Table 1). In particular, cod liver and flaxseed oils are rich in omega-3 polyunsaturated fatty acids; whereas the fatty acid composition of corn, peanut, and sunflower is mainly represented by oleic and linoleic acid, followed by palmitic acid. By contrast, castor oil shows a peculiar composition being rich in ricinoleic acid, conferring a higher viscosity and dielectric constant (Table 1). The dielectric constants of the other oils followed the order: flaxseed > cod liver > sunflower > corn > peanut oil > MCT [32]. Except for cod liver oil, whose heterogeneous fatty acid profile does not allow for a clear prediction of dielectric properties, this order is related to the content of linoleic acid [35].

### 3.3. Oleogel Properties

The absorption of the different oils into the WP aerogel particles led to semi-solid and plastic oleogels, whose shape could be easily modelled, as shown by the images reported in Table 2. After centrifugation two well-separated phases were obtained: the pellet containing the oleogel, and the supernatant, represented by the excess oil, which showed no apparent residues of aerogel particles. The only exception was castor oil, for which a clear phase separation was not obtained since WP aerogel particles remained dispersed in the oil. As a result, a soft material was obtained (Table 2). In the other cases, oils were effectively turned into materials presenting a peculiar plastic and deformable texture, whose colour was influenced by the original oil colour (Table 2). In all cases, the aerogel particles were able to absorb oil about four times their weight, leading to loaded systems showing an oil amount around 80% (Table 2). These data are in agreement with the findings of our previous study [26]. Such systems resulted physically stable for at least 5 months after preparation; during this time, in fact, no visible oil separation was observed.

**Figure 2:**
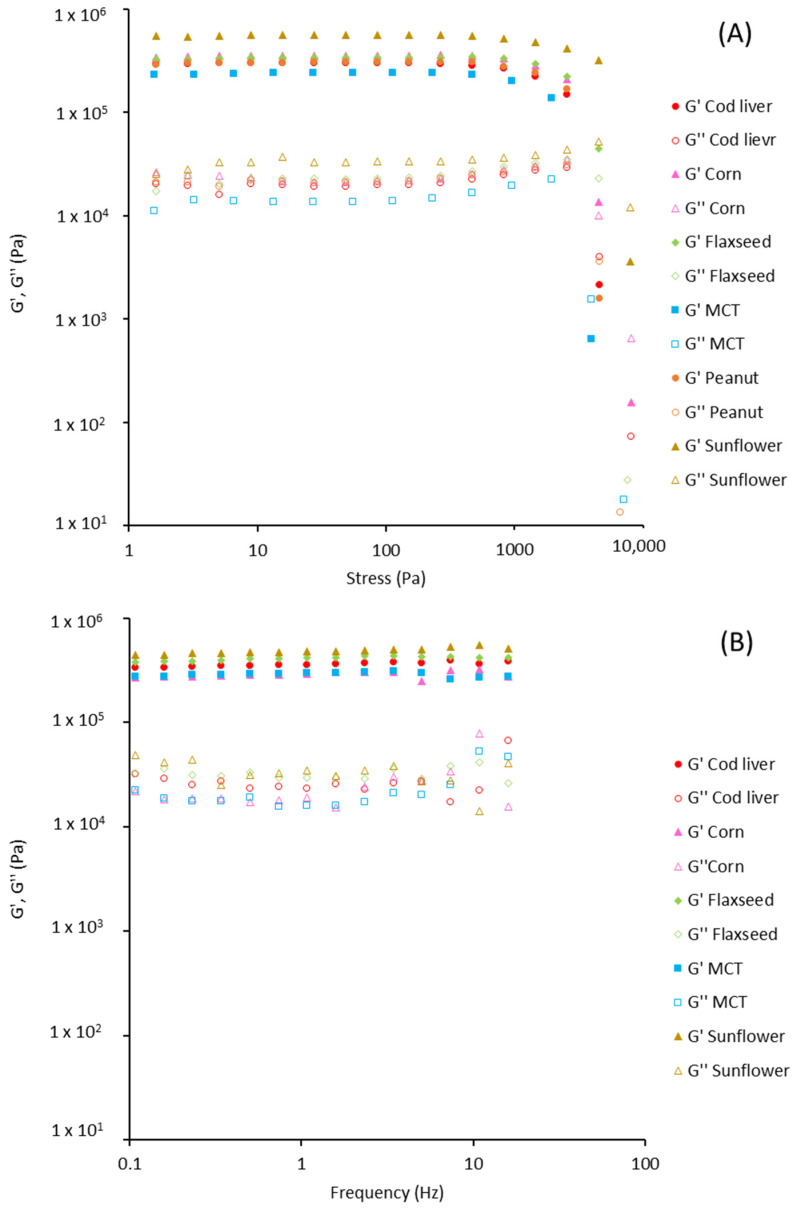
Dependence on the stress (**A**) and frequency (**B**) of G′ (solid symbol) and G″ (empty symbol) moduli of oleogels produced with different oils.

To better study the plastic properties of the obtained materials, rheological measurements were conducted (Figure 2, Table 3). The castor oil-derived system was not analysed, due to lack of structuration (Table 2). All the remaining samples behaved as gels with G′ higher than G″ (Figure 2). Figure 2A shows that the increase of stress up to about 800 Pa determined the drop of the LVR, with results comparable for all the samples (Table 3). G′ and G″ of oleogels in the frequency range from 0.1 to 20 Hz resulted independent on the applied frequency (Figure 2B), thus accounting for a strong-gel behaviour. This was further confirmed by the low values of the loss tangent (tan δ = G″/G′), which is inversely related to the elastic behaviour of the system (Table 3). The yield resistance and elastic response of the oleogels are likely due to the network formed by the WP particles in the oil, accounting for a plastic and deformable structure [26,34]. The similar rheological values of the oleogels obtained with the different oils (Table 3) highlighted a minor effect of the oil type on the networking ability of WP aerogel particles. Interestingly, the rheological parameters of these systems, as well as their lipid content are comparable to those of traditional solid fats, such as palm oil shortenings and margarine, typically used as spreads or for the preparation of laminated bakery goods [36]. WP aerogel particles could thus be used to obtain oleogels with similar structure and composition from a wide variety of different oils. This represents a key difference as compared to direct oil gelation strategies, in which the rheological features and stability of the final oleogels are strongly affected by the nature of the used oil, which affects the supramolecular organization of lipophilic building blocks [30,31,32,37]. A change in the mechanical properties with the oil composition has also been reported for oleogels obtained by indirect methods. For example, Wang et al. [33] applied the emulsion-templated method to obtain oleogels from a W/O emulsion structured using saturated monoglycerides. In this study, oleogel structure resulted affected by the oil type, due to the modification of oil-gelator interactions. Similarly, de Vries et al. [34] have demonstrated that the mechanical properties of WP particle-based oleogels prepared via an indirect oil gelation procedure, based on solvent exchange steps, are also influenced by the used oils, due to the interference in particle networking.

Confocal microscopy allowed further investigation into the effect of the oil characteristics on oleogel structural features (Table 2). Independently of the oil, the network was formed by highly porous protein particles able to absorb the oil within pores as well as to hold it in the inter-particle spaces. Based on the relatively high macroporosity of the WP aerogel particles (Figure 1), it can be concluded that the oil is efficiently absorbed into macropores, while mesopores have only a limited effect on oil structuring. The strength of the network formed by protein aerogel aggregates in oil has been shown to be determined by the interplay between particle–particle and particle–oil interactions. The predominance of particle–particle interactions would result in a stronger network, while larger particle–oil interactions would weaken the network [34]. Given the generally hydrophilic nature of whey protein aerogels [38,39,40], an increase of oil polarity would result in an increase of particle–oil interactions and thus in a softer texture. This is the case of castor oil, which contains ricinoleic acid, presenting a hydroxyl group in the fatty acid chain, which probably interacted with WP aerogel particle surface, hindering the formation of a strong network among particles [34].

**Figure 3:**
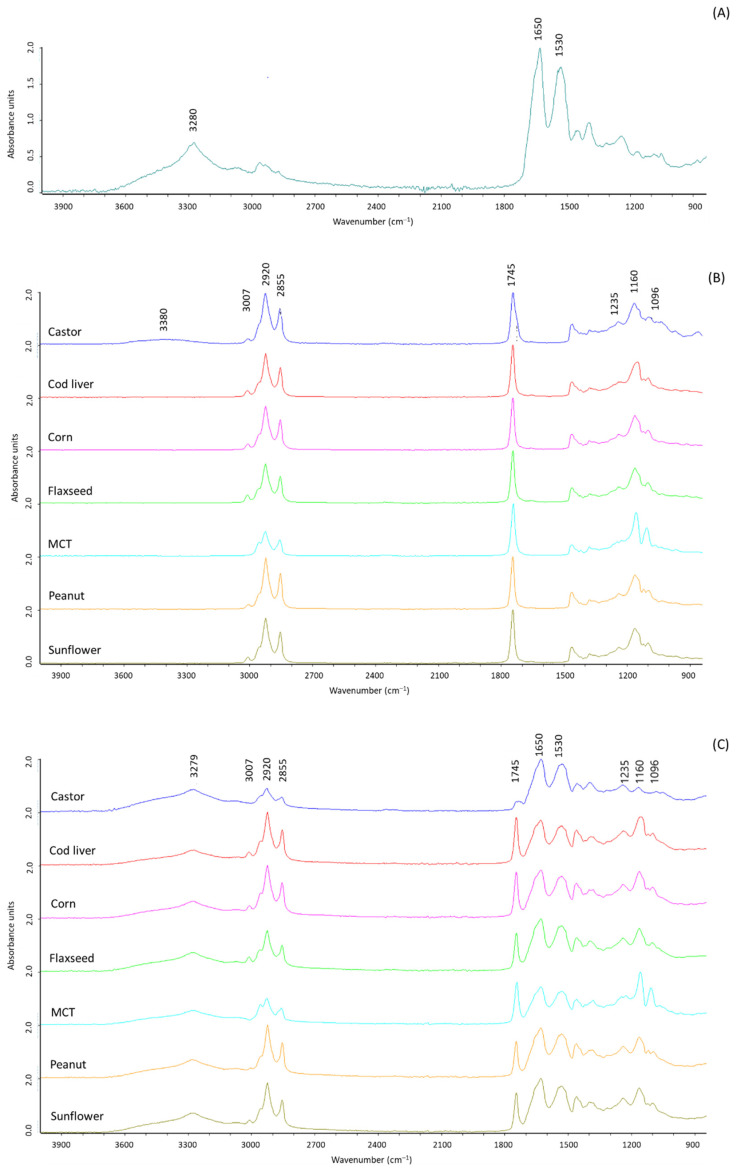
FTIR spectra of whey protein aerogel particles (**A**), oils (**B**,**C**) oleogels.

Based on these results, WP particles are able to structure liquid oil mainly due to physical phenomena, involving capillary absorption in the aerogel pores and weak hydrophilic inter–particle interactions. This was confirmed by FTIR analysis (Figure 3). The FTIR spectra of the aerogel particles (Figure 3A) showed the typical peaks of proteins, with a broad intense peak at around 3280 cm^−1^, corresponding to the N–H stretching vibration and bands at 1650 and 1530 cm^−1^, which correspond to amides I and II, respectively [41]. The spectra of the oils (Figure 3B) showed the typical bands of triglycerides contained in most edible oils [42]. The peaks at 3007, 2920 and 2855 cm^−1^ are attributed to = C−H stretching vibration, and C−H stretching vibrations of methylene and methyl groups, respectively [42]. The band at 1745 cm^−1^ is attributed to C = O double bond stretching vibration, and the peaks at 1235, 1160 and 1096 cm^−1^ are associated with deformation and bending of C−H and stretching vibration of C−O [43,44]. Castor oil also showed a broad band in the region 3600-3200 cm^−1^, related to the presence of hydroxyl groups of ricinoleic acid [45]. The FTIR profiles of the oleogels (Figure 3C) presented the peaks detected in proteins (Figure 3A) and oils (Figure 3B), confirming that no strong chemical interactions were formed between the aerogel scaffold and the liquid oil. Moreover, the independence of oleogel structuring on the oil type was further confirmed by the absence of differences in the spectra of the oleogels produced with the different oils (Figure 3C).

## 4. Conclusions

Whey protein aerogel particles can be used to obtain oleogels based on the ability to absorb oil in their porous structure and form a network among particles. The particles used in this study presented a relatively low internal surface area due to the used pH (near the isoelectric point) so that a further improvement in the oleogelation capacity could be expected by changing the pH conditions.

The presented oleogelation approach would offer various advantages. Firstly, the developed oleogels may fall into clean labels as they are simply composed of whey proteins and oil, which are commonly used food ingredients and widely accepted by consumers. Secondly, the development of oil oxidation is prevented since a cold oleogelation is performed by absorbing oil into the aerogel porous structure without any oil heating step. Finally, this approach is characterised by high versatility as the structuring properties of whey protein aerogel particles are not affected by the oil type, with the only exception of high-polarity oils.

Based on these characteristics, the developed oleogels can be considered optimal candidates for the delivery of lipophilic molecules while reducing saturated fat content. In this regard, the applicability of these oleogels in real food matrices should be assessed to understand their compatibility with other food components, as well as their performances during food processing and storage. The developed structures could also behave as peculiar delivery systems for health-promoting lipophilic components not only during food processing and storage, but also upon its consumption, possibly modulating their release in the gastrointestinal tract.

## Figures and Tables

**Table 1:** Viscosity and dielectric constant (ε′, 25 °C) of castor, cod liver, corn, flax-seed, medium-chain triacylglycerols (MCT), peanut, and sunflower oil.

Oil	Castor	Cod Liver	Corn	Flax Seed	MCT	Peanut	Sunflower
Viscosity (Pa s)	1.010 ± 0.002 ^a^	0.063 ± 0.003 ^b,c^	0.064 ± 0.003 ^b,c^	0.053 ± 0.003 ^c^	0.030 ± 0.002 ^d^	0.080 ± 0.001 ^b^	0.077 ± 0.014 ^b^
ε′ *	4.55	3.20	3.15	3.27	3.75	3.10	3.18

* Data from Valoppi et al. [32]; ^a,b,c,d^ In the same raw, means indicated by different letters are statistically different (*p* < 0.05).

**Table 2:** Appearance, optical and confocal microscopic structure, and oil content of oleogels produced by absorption of different oils by whey protein aerogel particles. Green = oil; red = proteins.

Oil	Appearance	Optical Micrograph	Confocal Micrograph	Oil Content (%, *w*/*w*)
Castor	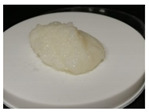	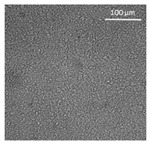	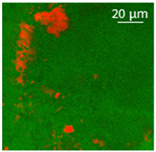	n.d.
Cod liver	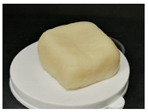	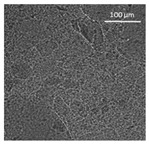	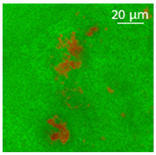	78.6 ± 0.7 ^a^
Corn	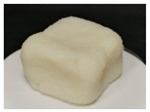	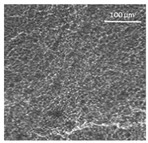	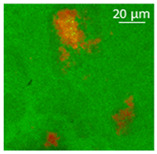	80.9 ± 0.4 ^a^
Flaxseed	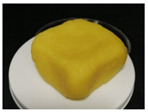	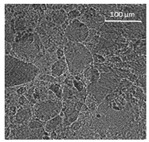	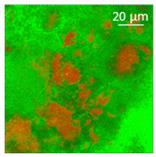	81.4 ± 1.6 ^a^
MCT	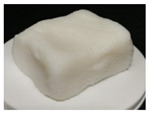	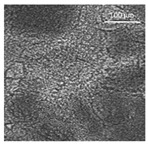	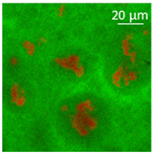	82.6 ± 0.6 ^a^
Peanut	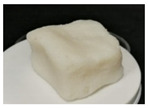	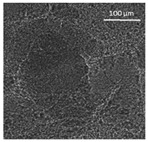	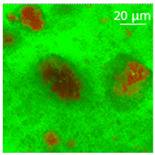	79.2 ± 1.8 ^a^
Sunflower	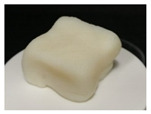	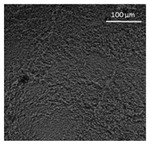	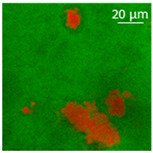	82.2 ± 3.7 ^a^

n.d., not determined; ^a^ in the same column, means indicated by different letters are statistically different (*p* < 0.05).

**Table 3:** Rheological parameters (G′, G″ compared at 1 Hz, critical stress, tan δ) of WP aerogel particles loaded with different oils.

Oil	G′ (Pa) × 10^5^	G″ (Pa) × 10^5^	Critical Stress (Pa)	Tan δ
Cod liver	3.3 ± 0.4 ^a^	0.22 ± 0.02 ^a^	793.3 ± 59 ^a^	0.065 ± 0.002 ^a,b^
Corn	3.0 ± 0.4 ^a^	0.21 ± 0.04 ^a^	848.9 ± 30 ^a^	0.073 ± 0.006 ^a^
Flaxseed	4.3 ± 0.1 ^a^	0.29 ± 0.02 ^a^	873.7 ± 48 ^a^	0.069 ± 0.002 ^a^
MCT	3.5 ± 0.4 ^a^	0.19 ± 0.02 ^a^	884.5 ± 3.8 ^a^	0.054 ± 0.003 ^b^
Peanut	3.9 ± 0.1 ^a^	0.25 ± 0.08 ^a^	849.2 ± 4.9 ^a^	0.074 ± 0.007 ^a^
Sunflower	4.4 ± 0.5 ^a^	0.26 ± 0.09 ^a^	818.7 ± 13 ^a^	0.067 ± 0.005 ^a^

^a,b^ In the same column, means indicated by different letters are statistically different (*p* < 0.05).

## Data Availability

Raw data were generated at the Department of Agricultural, Food, Environmental and Animal Sciences of the University of Udine and at the Institute of Thermal Separation Processes of the Hamburg University of Technology. Derived data supporting the findings of this study are available from the corresponding author S.C. on request.

## Supplementary Materials

The following are available online at https://www.mdpi.com/article/10.3390/polym13234063/s1, Figure S1: Original pictures of aerogel microstructure obtained by SEM with the process details and scale bar.
