# Supplementary material for: Conversion of Whey Protein Aerogel Particles into Oleogels: Effect of Oil Type on Structural Features
Source: Polymers (Basel). 2021 Nov 23;13(23):4063. doi: 10.3390/polym13234063 (PMC8659083; doi:10.3390/polym13234063)
Supplement: Supplementary file 1 [file polymers-13-04063-s001.zip › polymers-1458104-supplementary.pdf]

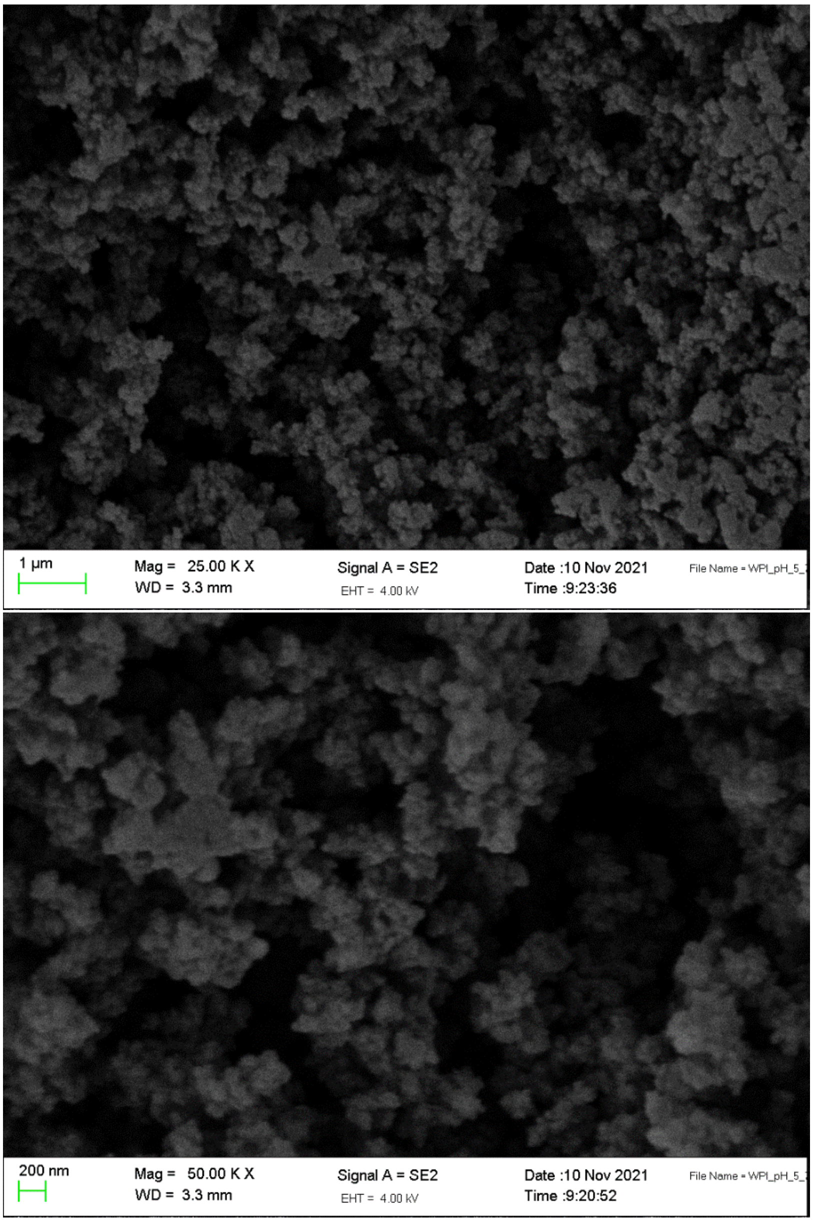

Figure S1. Original pictures of aerogel microstructure obtained by SEM with the process details and scale bar
